# Modeling the Three-Dimensional Bioprinting Process of β-Sheet Self-Assembling Peptide Hydrogel Scaffolds

**DOI:** 10.3389/fmedt.2020.571626

**Published:** 2020-10-15

**Authors:** Irene Chiesa, Cosimo Ligorio, Amedeo F. Bonatti, Aurora De Acutis, Andrew M. Smith, Alberto Saiani, Giovanni Vozzi, Carmelo De Maria

**Affiliations:** ^1^Research Center ‘E. Piaggio’, University of Pisa, Pisa, Italy; ^2^Department of Ingegneria dell'Informazione, University of Pisa, Pisa, Italy; ^3^Department of Materials, The University of Manchester, Manchester, United Kingdom; ^4^Manchester Institute of Biotechnology, The University of Manchester, Manchester, United Kingdom

**Keywords:** self-assembling peptide hydrogel, finite element modeling, extrusion-based 3D bioprinting, printability, scaffolds

## Abstract

Extrusion-based three-dimensional (3D) bioprinting is nowadays the most efficient additive manufacturing technology to fabricate well-defined and clinical-scale relevant 3D scaffolds, exploiting soft biomaterials. However, trial and error approaches are usually employed to achieve the desired structures, thus leading to a waste of time and material. In this work, we show the potential of finite element (FE) simulation in predicting the printability of a biomaterial, in terms of extrudability and scaffold mechanical stability over time. To this end, we firstly rheologically characterized a newly developed self-assembling peptide hydrogel (SAPH). Subsequently, we modeled both the extrusion process of the SAPHs and the stability over time of a 3D-bioprinted wood-pile scaffold. FE modeling revealed that the simulated SAPHs and printing setups led to a successful extrusion, within a range of shear stresses that are not detrimental for cells. Finally, we successfully 3D bioprinted human ear-shaped scaffolds with *in vivo* dimensions and several protrusion planes by bioplotting the SAPH into a poly(vinyl alcohol)–poly(vinyl pyrrolidone) copolymer, which was identified as a suitable bioprinting strategy by mechanical FE simulation.

## Introduction

Over the past decades, additive manufacturing (AM) technologies have evolved from simple rapid prototyping tools to life-saving patient-specific fabrication methodologies and have been exploited in many research areas such as tissue engineering (TE) (1–8).

The application of AM processes to the fabrication of three-dimensional (3D) structures by the deposition and assembling of living and/or non-living biomaterials with an established organization is referred to as bioprinting (9–11). In contrast with conventional techniques (e.g., solvent casting, particulate leaching, and freeze drying), bioprinting allows to closely control the scaffold fabrication in terms of material composition and geometry, including shape, distribution, and interconnectivity of internal pores. Moreover, bioprinting can be used to fabricate 3D structures from the volumetric information of a damaged tissue or organ from patients' medical images (1, 11).

Among bioprinting technologies, extrusion-based 3D bioprinting, which dispenses material through microscale nozzles, is the most efficient approach to fabricate well-defined 3D scaffolds with clinically relevant size, within a realistic time frame (5, 10, 12, 13). This technology is also compatible with a broad range of biomaterial inks/bioinks with viscosities ranging from 30 mPas to 6 × 10^4^ Pas (14) and at a reasonable cost (2, 15). Multiple physical–chemical material requirements have been identified as favorable and necessary for extrusion-based 3D bioprinting (1, 5), which narrow down the range of potential biomaterial inks/bioinks that can be processed with this technique. The most important features of an ideal biomaterial ink/bioink are fast gelation after extrusion, high viscosity, high yield stress, shear-thinning behavior, and high elastic modulus (>100 kPa) (3, 10, 16).

Hydrogels are commonly used materials for soft TE as they recapitulate several features of native extracellular matrix, such as its highly hydrated nature and fibrillar architecture, as well as provide a template for tissue ingrowth and cell proliferation (17). In addition, tuning biological and mechanical properties of these soft biomaterials is usually straightforward and bioactive compound (e.g., drugs, growth factors, and peptide sequences) can easily be grafted to the material network to modulate cellular behaviors and create multifunctional materials (10, 18–20). Self-assembling peptide hydrogels (SAPHs) are gaining an increasing interest in TE, due to their versatile bottom-up design, biocompatibility, and ease of functionalization. Indeed, by exploiting the 20 natural amino acids as a library of building blocks, several designs of peptide sequence, which self-assemble into nanofibers under a range of different stimuli, such as pH, enzymes, and temperature, have been developed (21). Above a critical gelation concentration, those nanofibers associate, entangle, and entrap a large amount of water leading to the formation of supramolecular swollen 3D networks, i.e., hydrogels.

Four main molecular designs have been proposed for the formulation of peptide-based hydrogels: amphiphilic (22), β-sheet forming (20), short aromatic (23), and alpha-helices/coiled-coil forming peptides (24, 25). In this work, we investigated a class of β-sheet forming peptides based on the alternation of hydrophilic and hydrophobic amino acids (26) that self-assemble into cross-β-sheet-fibers and nanofibrillar hydrogels. This class of hydrogels has been shown to be suitable for the culture of a range of cells including nucleus pulposus, osteoblasts, chondrocytes, and mesenchymal stem cells. Their low immunogenic response and excellent biocompatibility make these hydrogels attractive in drug delivery and screening, tissue regeneration, and injectable cell therapy applications (27–30).

Recently, peptide-based hydrogels have started to be explored for bioprinting (31). Notably, they meet several key requirements: they are shear-thinning and able to recover after shear, which means that no additional crosslinkers are required (32, 33). This reduces the fabrication times and the amount of toxic by-products. In addition, peptide self-assembly can be modulated by acting on several intrinsic [e.g., peptide sequence (34) and concentration] and extrinsic factors [e.g., pH, temperature, and salt concentration (35)].

In this scenario, finite element (FE) analysis is a powerful tool to investigate how those factors actually influence the extrusion-based bioprinting process of SAPHs, thus decreasing the trial and error experiments and material waste (13, 36–38). Simulating the material flow in the needle during the extrusion process allows to understand the relation between needle size, printing parameters, and material properties (13) and therefore facilitates the optimization of the printing parameters and the biomaterial ink/bioink formulation. Emmermacher et al. (13), for example, analyzed the material flow in a conical printing needle for different applied pressures and needle outlet diameters. The authors summarized the results in a material-specific chart, which allows the user to easily select printing parameters (needle diameter and applied pressure) to get the desired mass flow rate and the maximum shear stress.

By FE simulations, viscous forces that arise inside the needle during the extrusion can be also derived and compared with shear stress values that are compatible with cell survival (13, 39, 40). Gaining this type of information is extremely important for the 3D bioprinting of living cells and for the design of new bioinks. In addition, FE simulations can also predict the mechanical behavior of scaffolds in terms of mechanical stability over time (41, 42). For instance, Soufivand et al. (42) tuned and predicted the mechanical behavior of a 3D-printed polycaprolactone (PCL) scaffold based on its inner geometries by FE analysis. Similarly, Koh et al. (41) identified the optimum material properties of a scaffold for cartilage regeneration by an FE model under gait cycle conditions.

3D bioprinting of volumetric structures with a well-defined geometry from soft biomaterials, including SAPHs, represents a significant challenge, due to their weak mechanical properties and low viscosity. This challenge has led to the development of new and innovative bioprinting strategies using external sacrificial support materials, whose goal is to stabilize the printed structures (43–45).

In this context, using bioplotting strategies, a biomaterial ink/bioink is extruded directly into a shear-thinning and easy-to-remove sacrificial material with a high yield stress. This material is not a part of the manufactured structure but acts as a temporary support matrix during the bioprinting process to stabilize the bioprinted structure and avoid its premature collapse before the end of the crosslinking reaction/process (1, 9, 44). We recently used this bioplotting technique with pluronic acid F127 (46) to 3D bioprint wood-pile scaffolds made of a mixture of gelatin type A, nanohydroxyapatite, and genipin. By using bioplotting, we prevented the structure to collapse, allowing the retention of geometric features, such as scaffold axial and longitudinal pores.

In the present work, we tested the suitability of a β-sheet forming peptide FEFKFEFKK (F9) hydrogel for 3D bioprinting of volumetric scaffolds. The F9 hydrogel rheological properties were evaluated, and subsequently, FE simulations were carried out to establish the bioprinting parameters and to get an insight into scaffold mechanical stability over time after the actual deposition. Finally, an ear-shaped scaffold with clinically relevant dimensions was 3D bioprinted *via* bioplotting by exploiting a poly(vinyl alcohol)–poly(vinyl pyrrolidone) (PVA-PVP) copolymer as sacrificial support material.

## Materials and Methods

### Hydrogel Preparation

FEFKFEFKK peptide (where F = phenylalanine, E = glutamic acid, and K = lysine) was purchased as hydrochloric acid salt from Biomatik Corporation (Wilmington, DE, Canada). Peptide purity, 97%, was confirmed in-house by reversed-phase high-performance liquid chromatography (HPLC). Depending on the final peptide concentration targeted (20 and 30 mg/ml), lyophilized peptide powder was dissolved in HPLC-grade water and pH adjusted to values ranging from 3.7 to 4.9, within the hydrogel gelation window, using a 0.5 M sodium hydroxide (NaOH) solution.

### F9 Hydrogel Rheological Characterization

Oscillatory shear rheometry was performed on a Discovery Hybrid 2 (DHR-2) rheometer (TA Instruments, USA) using a 20-mm parallel plate geometry with a gap size of 500 μm. Samples (200 μl each) were prepared by pipetting the hydrogel onto the rheometer stage before lowering the upper head to the desired gap size and left to equilibrate for 180 s at 25°C. To evaluate the viscoelastic behavior of the hydrogels, amplitude sweep tests were performed at 1 Hz, in the shear strain range of 0.01–1%, while frequency sweep tests were performed at 0.2% strain, within the linear viscoelastic region in the frequency range: 0.01–10 Hz. Finally, to assess the viscosity behavior under shear stress, flow sweep experiments were performed at 0.2% strain at 1 Hz in the shear rate range of 0.01–2,000 1/s. All measurements were repeated at least three times to ensure reproducibility.

### Sacrificial Support Material Rheological Characterization

PVA-PVP copolymer (ratio 1:1) (47) was used as sacrificial support material because it is water-soluble and therefore easy to remove from the F9 hydrogel. Its rheological properties were investigated. Oscillatory shear rheometry was performed using a HAAKE RheoStress 6000 (Thermo Scientific) with plate-cone (1° angle) measuring configuration. Samples were prepared as described in the previous section. Measurements were performed in the linear viscoelastic regime, which was initially determined by a shear sweep (0.1–100 Pa) at 25°C (printing temperature) and a frequency of 1 Hz. This initial test allows to analyze the storage modulus and the loss modulus in relation to the shear rate. Then, the viscosity curve was recorded at a fixed strain in the frequency range of 0.1–200 s^−1^ at 25°C. Additionally, the yield stress as function of shear rate was determined.

### Finite Element Analysis

#### Extrusion Process

**Figure 1:**
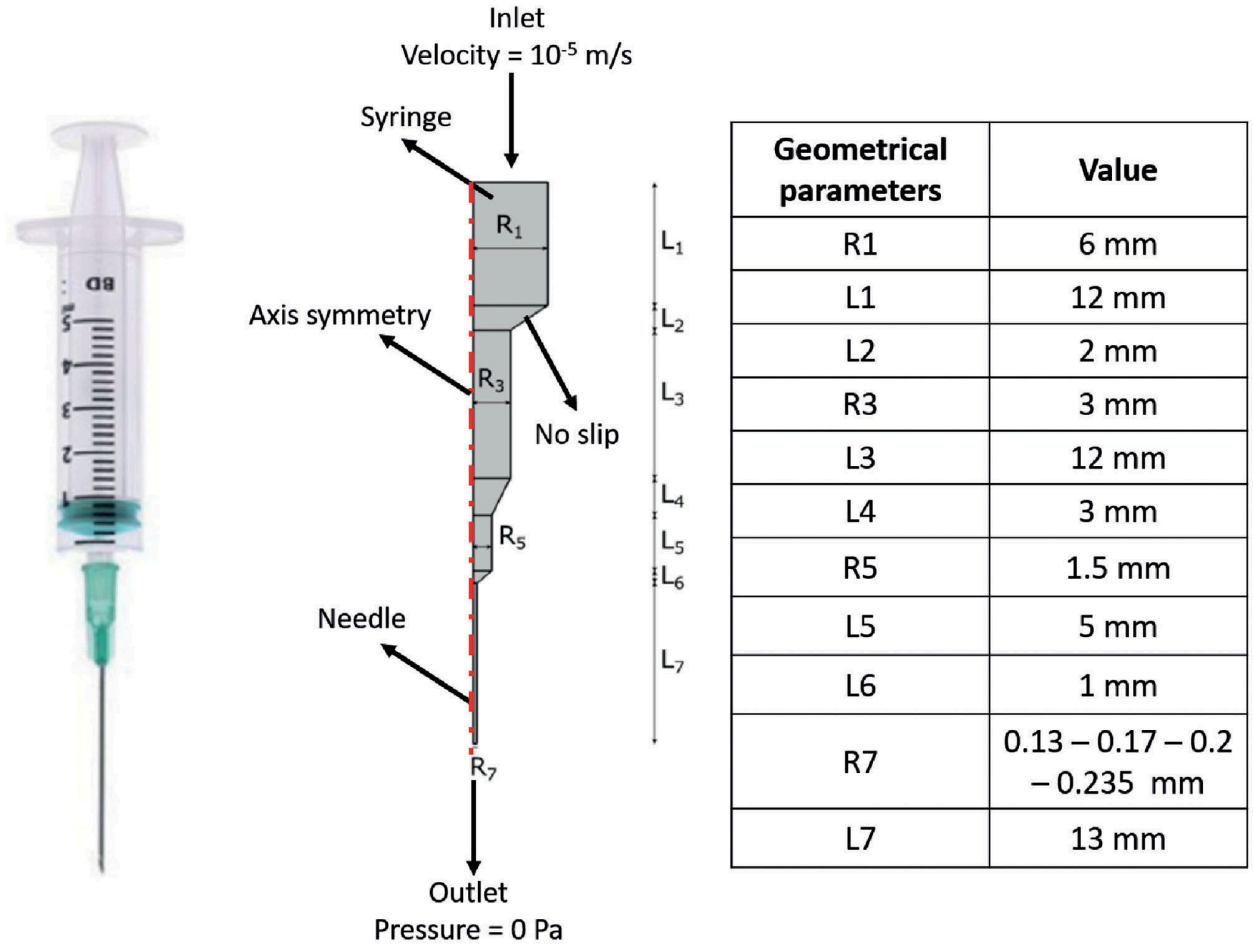
Schematic of the extrusion platform used in the finite element (FE) simulations. Geometrical dimensions are reported. A 2D axial symmetric model was applied, comprising a single domain (i.e., F9 hydrogel) and four different boundary conditions, which are reported in the figure.

FE models were implemented in Comsol Multiphysics (Comsol Inc., 5.3) to simulate the extrusion process of the F9 hydrogels, through a piston-driven 3D bioprinter comprising a 5-ml commercial syringe (Becton Dickinson) and a cylindrical nozzle (Figure 1), as key components of the extrusion system (46). Parametric models were implemented. Firstly, six different combinations of F9 hydrogel concentration and pH, reported in Table 1, were defined as domain in combination with a 0.4-mm needle. Then, needles with different radius R7 (0.13, 0.17, 0.2, and 0.235 mm, as reported in Figure 1) were simulated, keeping the domain properties constant, using those of the F9 hydrogel with the concentration equal to 20 mg/ml and pH equal to 3.7.

**Table 1:** Combinations of F9 hydrogel concentration and pH implemented in the simulations.

	**C = 20 mg/ml**			**C = 30 mg/ml**		
pH	3.7	4.8	4.9	3.7	4.8	4.9
K [Pas^n^]	5.78	13.46	16.83	13.91	21.18	41.98
n	0.12	0.05	0.02	0.04	0.02	0.02

*Flow consistency factors (K) and flow behavior index (n) were obtained by linear regression of rheological experimental data and implemented in finite element (FE) simulations*.

In all simulations, the non-Newtonian flow application mode in stationary conditions was used. A 2D axis-symmetric model was applied, consisting of a single domain, with density equal to 1,000 kg/m^3^. The F9 hydrogels are shear-thinning fluids; thus, in the simulations, they were described by the power law model of Ostwald and de Waele (Equation 1) (48):


(1) $$\begin{array}{r} \eta =K\;*\;{\left(\overset{∙}{\gamma }\right)}^{n-1} \end{array}$$

where $\overset{∙}{\gamma }$ is the shear strain (s^−1^), *K* is the flow consistency factor (Pas^n^), and *n* is the flow behavior index.

For each F9 hydrogel concentration and pH that were simulated, *K* and *n* were derived from rheological data (*F9 Hydrogel Rheological Characterization*). Briefly, the related shear rate and viscosity data were transformed using the logarithm function, and then, a linear fitting was applied to get *K* and *n*, which are reported in Table 1. The *R*^2^ values for these fittings were >0.95 for all the concentrations and pH values.

Boundary conditions used in the simulations are indicated in Figure 1. The piston-driven extrusion was simulated by imposing a constant velocity as inlet (10^−5^ m/s), derived from our experimental setup (*Bioprinting of Scaffolds*), the boundaries on the axis of symmetry were set as “axial symmetry,” the tip of the needle R7 was set as outlet with no pressure, and the remaining external boundaries were set as “no slip” condition. A triangular mesh, controlled by the physics, was used in all the simulations. Mesh statistics are shown in Table 2.

**Table 2:** Mesh statistics of fluid dynamic (Extrusion Process) and mechanical simulations (Analysis of Scaffold Deformation After Printing).

	**Fluid dynamics**	**Mechanical without support**	**Mechanical bioplotting**
Maximum element size (m)	2.1 * 10^−4^	1.2 * 10^−4^	1.86 * 10^−4^
Minimum element size (m)	6 * 10^−6^	3.78 * 10^−7^	6.28 * 10^−7^
Maximum element growth rate	1.13	1.25	1.25
Curvature factor	0.3	0.25	0.25
Number of elements	16,290	602	536
Minimum quality	0.3617	0.6886	0.5848
Average quality	0.8673	0.8913	0.8662

#### Pressure Drop Analysis With Different Rheological Models

Pressure drop along the extrusion system (syringe + needle) was deeply analyzed in order to investigate the approximation error connected to different rheological models:

1. Model 1: Newtonian model with viscosity equal to K.
2. Model 2: Newtonian model with viscosity set as F9 hydrogel's viscosity at the average shear strain. Firstly, the value of the average velocity in the needle (*Velocity*_*outlet*_) was derived by Equation (2), which describes the mass conservation throughout the extrusion system (needle + syringe):
  (2) $$\begin{array}{r} Velocity_{inlet\;}\;*\;{Area\;}_{syringe}=\;Velocity_{outlet}\;*\;{Area\;}_{needle\;} \end{array}$$
  where the *Velocity*_*inlet*_ was set as the one imposed as inlet in the simulation (10^−5^ m/s). Then, the average shear strain ($\bar{\overset{∙}{\gamma }}$) was evaluated as Equation (3).
  (3) $$\begin{array}{r} \bar{\overset{∙}{\gamma }}=\;\frac{8\;*\;Velocity_{outlet}\;}{Needle\;Diameter\;} \end{array}$$
  Then, the viscosity was obtained by Equation (1) using *n* and *K* from Table 1.
3. Model 3: Power law model, as described in the previous section.

These three rheological models were simulated using the same FE scheme (F9 hydrogel pH values and concentrations, syringe geometry, and boundary settings) described in the previous section and summarized in Figure 1 and Table 1. The difference respect to the power law model (model 3), assumed as reference, was evaluated according to Equation (4):


(4) $$\begin{array}{l} Modelling\;error=\frac{Pressure_{model\_i}-\;Pressure{\;}_{model\_3}}{Pressure_{model\_3}}\cdot 100,\\ \;with\;i\in \left\{1,2\right\} \end{array}$$

#### Analysis of Scaffold Deformation After Printing

**Figure 2:**
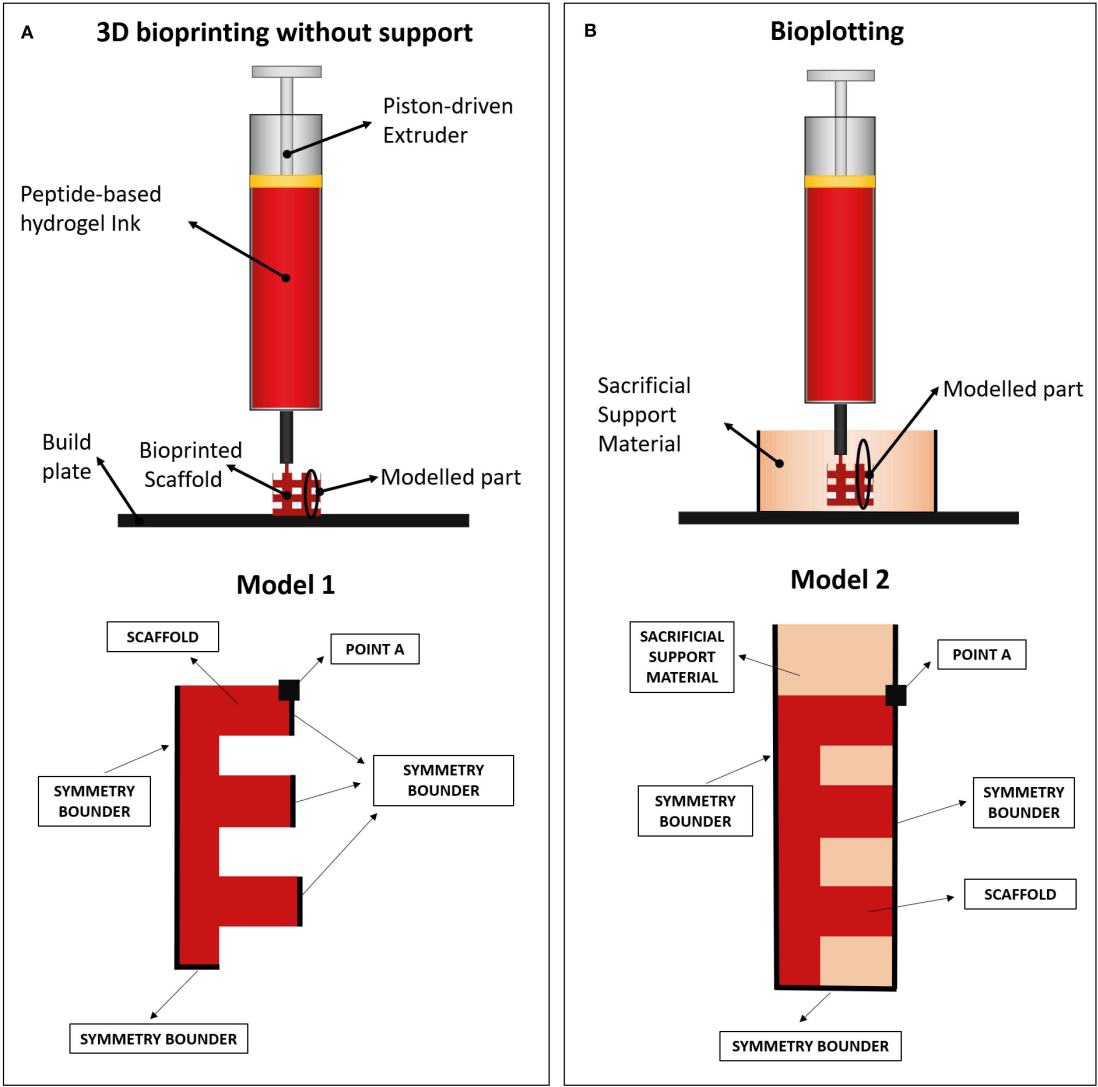
**(A)** Wood-pile scaffold bioprinted directly on the build plate (bioprinting without support) and schematic of subdomains and boundary conditions used in its model (model 1). In model 1, a single domain representing the scaffold is used (red). **(B)** Wood-pile scaffold bioprinted inside a sacrificial support material (bioplotting) and schematic of subdomains and boundary conditions used in its model (model 2). In model 2, two subdomains were used, representing the scaffold (red) and the sacrificial support material (orange).

FE models were implemented in Comsol Multiphysics (Comsol Inc., 5.3) to evaluate the collapse of a 3D-bioprinted wood-pile scaffold due to gravity force. Two different extrusion-based 3D bioprinting strategies were implemented and compared: (1) scaffold bioprinted without sacrificial support material and (2) scaffold bioprinted into a sacrificial support material (i.e., bioplotting) (Figure 2A).

The structural mechanical module for plane strain analysis was used, implementing a 2D model (thickness of 1 mm). A simplified model of a wood-pile scaffold (Figure 2B, red) was used as domain. Model 1 included a single domain, representing the scaffold, with viscoelastic properties, subjected to the volumetric load of its own weight. The Kelvin–Voigt model was used, characterized by a Hookean elastic spring and a Newtonian dumper in parallel (49, 50). Lumped parameters were obtained from the rheological characterization of the hydrogel (*F9 Hydrogel Rheological Characterization*). Briefly, the Newtonian dumper was set equal to the F9 hydrogel viscosity at low shear strain (η_0_), whereas the Hookean elastic spring constant (E) was obtained from storage modulus of the F9 hydrogel at low shear strain ($G_{0}\prime$) by Equation (5):


(5) $$E=2\cdot \left(1+\upsilon \right)\cdot G_{0}\prime$$

where υ is the Poisson ratio and was set equal to 0.49 due to the high content of water in the F9 hydrogels.

A different model for each combination of F9 hydrogel pH and concentration was implemented; η_0_, $G_{0}^{\prime }$, and E were used in each model as shown in Table 3. Scaffold geometrical dimensions were chosen in accordance with the slicing of a cube carried out with the following printing parameters: layer height equal to 0.55 mm, infill equal to 30% (inter-fiber distance = 2.57 mm), and needle size equal to 0.77 mm. The density of the material was kept constant at 1,000 kg/m^3^ due to its high water content.

**Table 3:** Lumped parameters of the Kelvin–Voigt model used in the mechanical simulation for both the F9 hydrogels and the PVA-PVP copolymer.

		**η_0_ (Pas)**	**G′_0_ (Pa)**	**E (Pa)**
F9 hydrogel	C = 20 mg/ml - pH 3.7	231.4	15	30
	C = 20 mg/ml - pH 4.8	895	55.5	111
	C = 20 mg/ml - pH 4.9	1,124	130	260
	C = 30 mg/ml - pH 3.7	981.4	64.5	129
	C = 30 mg/ml - pH 4.8	1,848.4	125	250
	C = 30 mg/ml - pH 4.9	5,237.4	369	738
Sacrificial support material (PVA-PVP copolymer)		1,500	500	1,000

*PVA, poly(vinyl alcohol); PVP, poly(vinyl pyrrolidone)*.

In the second model, the PVA-PVP copolymer was introduced as sacrificial support material. Therefore, a second subdomain was added, representing the support material itself (Figure 2B, orange). This subdomain has viscoelastic properties, described by the Kelvin–Voigt model. Its lumped parameters were obtained from its rheological characterization (section Sacrificial Support Material Rheological Characterization) as described for the F9 hydrogels: η_0_, $G_{0}^{\prime }$, and E are shown in Table 3. Its density was estimated equal to 1,000 kg/m^3^ due to its high water content.

In all models, a transient analysis between 0 and 200 s was used to evaluate the scaffold collapse over time. The gravity force, which causes scaffold collapse, was applied as a volumetric load in the domains. Boundary conditions are showed in Figure 2B. In particular, the symmetrical repetition of the “unit cell” of the woodpile scaffold was modeled using the symmetry conditions at the boundaries. A triangular mesh, controlled by the physics, was used in all the simulations. Mesh statistics are shown in Table 2.

### Bioprinting of Scaffolds

**Figure 3:**
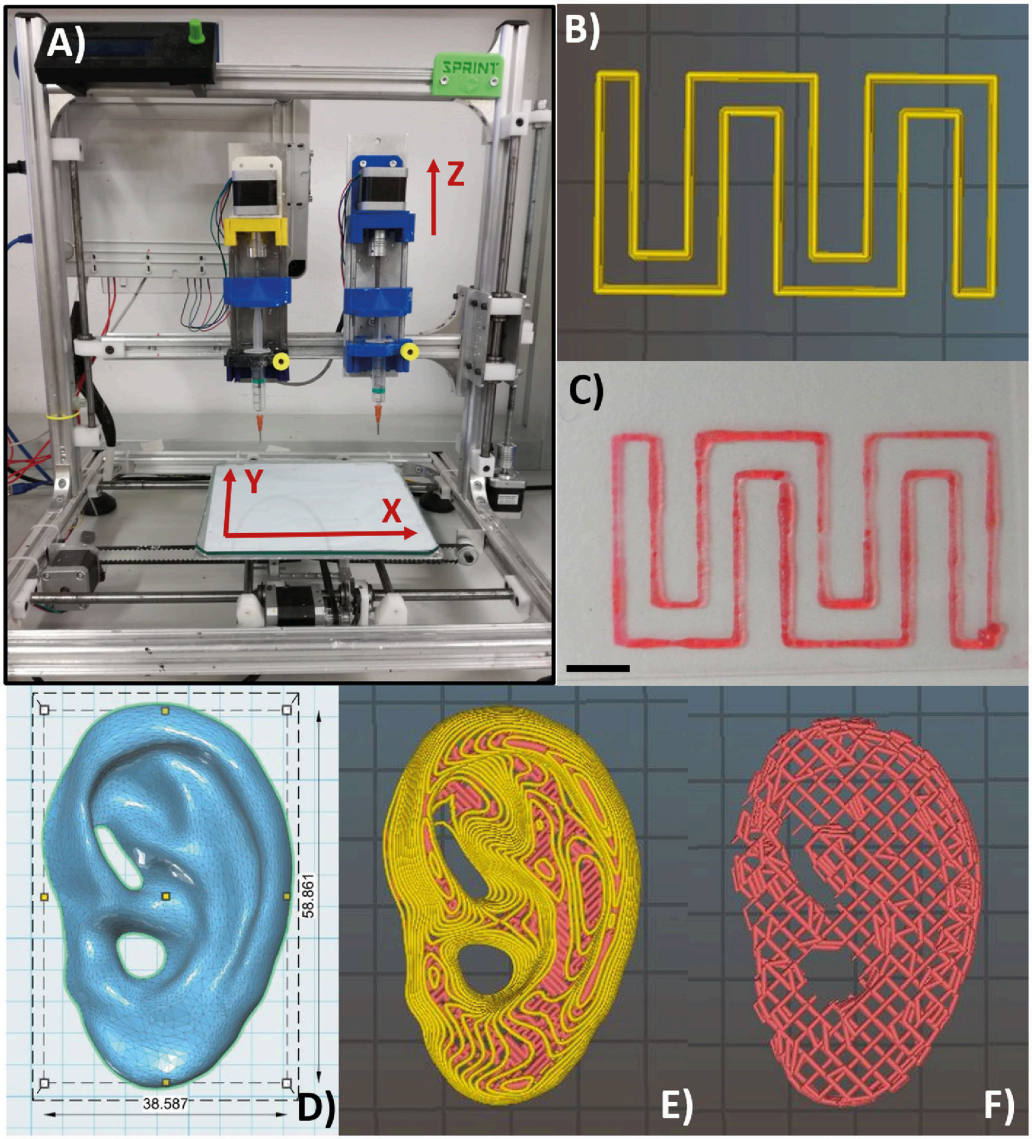
Piston-driven extruder 3D bioprinter used in all the bioprinting tests **(A)**. **(B,C)** Monolayer snake-like structure used for the bioprinted line characterization according to print speed and nozzle diameter. **(B)** Material deposition preview on Slic3r and **(C)** bioprinted monolayer structure. Notably, the printed structure has a high shape fidelity. Scale bar = 5 mm. **(D)** Human ear computer-aided design (CAD) model with *in vivo* dimensions used as an example to 3D bioprint the F9 hydrogel into complex structure with several protrusion planes. Feret dimension: 38.6, 58.9, 12.2 mm. **(E,F)** Preview of material deposition on Slic3r®. Starting from a single CAD file, three different models were tested. In the first one and in the second one **(E)**, perimeters (yellow lines) were included. In the third one **(F)**, perimeters were not included, and therefore, the internal wood-pile structure is clearly visible.

Bioprinting tests were carried out using a piston-driven extruder 3D bioprinter, developed at the research center “Enrico Piaggio” of the University of Pisa (Figure 3A) (46, 51). It has a 3D positioning system: the build plate moves along the X and Y axes, while the extruder moves along the Z axis. The 3D bioprinter has a piston driven extruder: the piston of a commercial 5-ml syringe (internal diameter equal to 12 mm) mechanically pushes the material through the nozzle as a continuous filament. The piston is actuated by a stepper motor (NEMA 17, length = 40 cm and Torque_max_ = 40 N cm) through a lead-screw mechanism (M5, pitch = 0.8 mm). Considering an efficiency of 0.5, the maximum force that the piston can apply can be derived from Equation (6) and is equal to 1,570 N.


(6) $$\begin{array}{r} Force_{\max}=\frac{Efficiency\;\cdot \;Torque_{\max}}{pitch} \end{array}$$

Considering the 80% of the maximum force (namely, 1,250 N), the maximum pressure that the piston can apply to the biomaterial ink can be derived from Equation (7) and is around 10 MPa.


(7) $$\begin{array}{r} Force_{piston}=Pressure\;drop\;*\;Area_{piston\;} \end{array}$$

In all the bioprinting tests, the F9 hydrogel, with C = 20 mg/ml and pH = 3.4, was diluted 1:0.01 with a 0.5 M NaOH (Sigma Aldrich) solution in deionized water (final pH = 4.9). A red food coloring dye was added to the hydrogel (20 μl/ml) for a better visualization of the bioprinted structures.

#### Bioprinted Line Characterization

A snake-like structure was designed with SolidWorks® (SolidWorks Corp., 2017) and imported in Slic3r® (Figure 3B) for the gcode generation. A 0.3-mm single layer was bioprinted (Figure 3C) testing three print speeds (7.5, 10, and 20 m/s) and three nozzle diameters (0.34, 0.4, and 0.47 mm) (nine combinations in total). Images of the printed lines were acquired with a brightfield microscope (Olympus AX70), and the line width was measured using ImageJ®. For each combination of print speed and nozzle diameter, 15 images were acquired and measured. The average value of the line width was calculated for each combination of print speed and nozzle diameter. The Pearson correlation coefficient was used to identify linear correlation between printed line width and nozzle diameter and between printed line width and print speed. GraphPad Prism (GraphPad Software Inc., 7) was used for these statistical analyses.

#### 3D Bioprinting of Complex Structure

According to the results of mechanical simulations, to successfully fabricate complex structures, the F9 hydrogel was 3D bioprinted into the PVA-PVP copolymer, which acts as a sacrificial support material (Figure 2A).

The computer-aided design (CAD) model of human ear (Figure 3D) with *in vivo* dimension (38.6 × 58.9 × 12.2 mm^3^) and several protrusion planes was downloaded from Thingiverse (52) and uploaded on Slic3r® for the gcode generation.

Three different models were bioprinted. Model 1 (Figure 3E) and model 2 have one perimeter (i.e., the outer contour of each single printed layer, which is shown in yellow in the printing preview) but different nozzle diameters (0.77 mm and 0.47 mm, respectively), whereas model 3 (Figure 3F) shared the same nozzle of model 1 but has no perimeters. Printing parameters are shown in Table 4. All models were bioprinted by bioplotting in the PVP-PVA copolymer inside a Petri dish. After the printing, the Petri dish, containing the bioprinted structure and the sacrificial support material, was put in deionized water for 48 h to dissolve the support material and to retrieve the bioprinted structures.

**Table 4:** Printing parameters used for each model.

	**Model 1**	**Model 2**	**Model 3**
Flow rate	150%	150%	150%
Print speed	7.5	7.5	7.5
Nozzle size (mm)	0.77	0.47	0.77
Layer height (mm)	0.55 (22 layers)	0.35 (34 layers)	0.55 (22 layers)
Fill density	30%	25%	30%
Perimeters	1	1	0
Solid infill threshold area (mm^2^)	70	70	0
Infill/perimeter overlap	15%	15%	0

## Results

### Rheological Characterization

#### F9 Hydrogel

**Figure 4:**
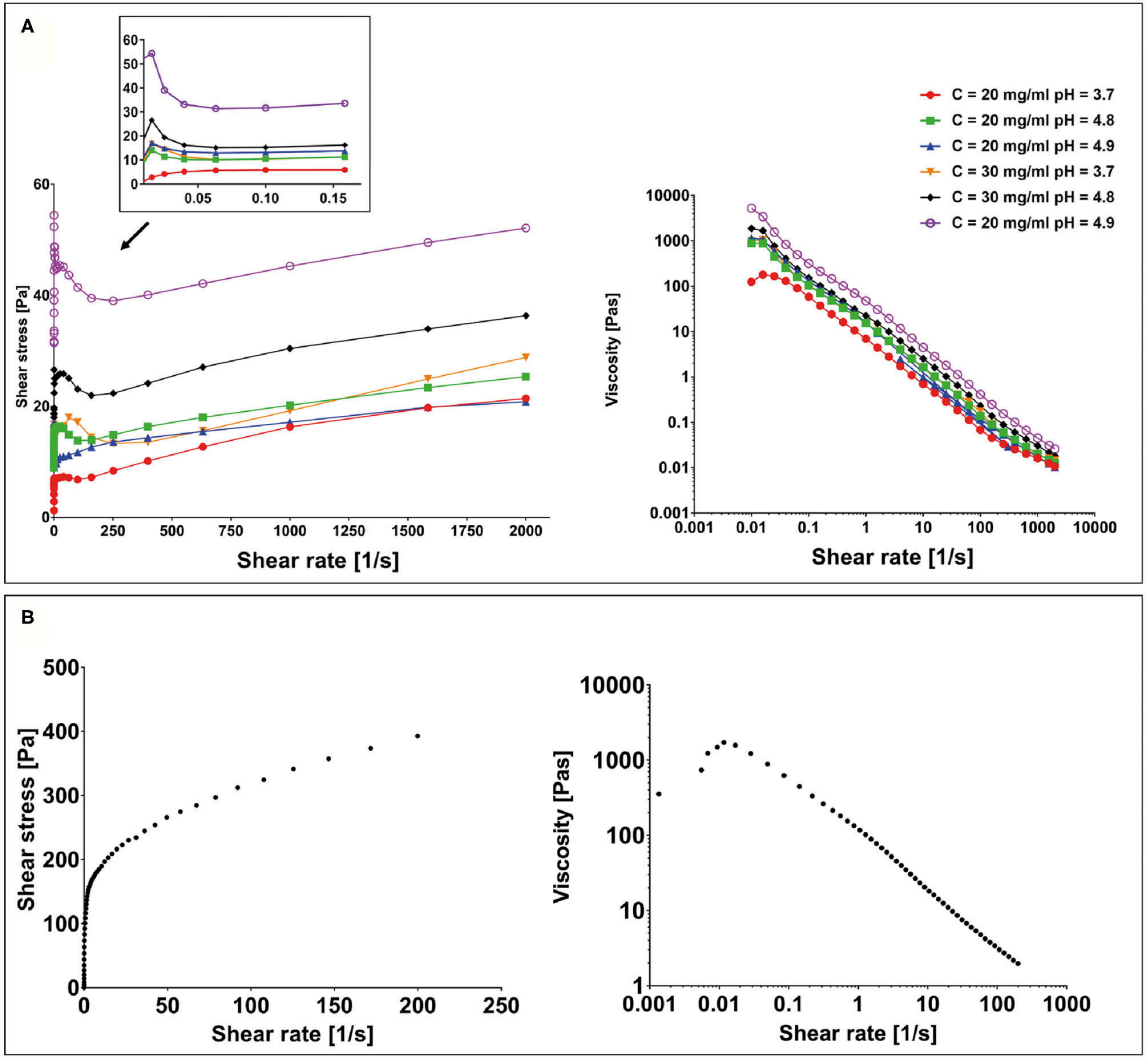
**(A)** Yield stress plot experiments performed on F9 hydrogels at 20 and 30 mg/ml at different pH values and viscosity curves. **(B)** Yield stress plot and viscosity curve performed on the poly(vinyl alcohol)–poly(vinyl pyrrolidone) (PVA-PVP) copolymer.

All hydrogel samples, tested at different pH values, showed a solid-like behavior across the shear strain (0.01–1%) and frequency range explored (0.01–10 Hz), having the storage modulus in each case at least one order of magnitude higher than the loss modulus (G′ > G″) (data not shown). To assess the printability of this peptide-based system, the rheological behavior of all F9 hydrogels was tested via oscillatory rheometry with flow sweep experiments. Results showed a general shear-thinning behavior with viscosity decreasing as shear rate increased for all the tested hydrogels. This trend was independent of peptide concentration (20 and 30 mg/ml) and pH values (3.7–4.9), which suggests a good ability of the material to undergo deformation within a large span of shear stresses, mimicking the process of hydrogel injection and printing (Figure 4A). Data revealed that a yield stress is visible at low shear rate for all the tested samples.

#### Sacrificial Support Material

To assess the suitability of the PVA-PVP copolymer as a sacrificial support material for bioplotting, its rheological behavior was tested via oscillatory rheometry with shear sweep (data not shown) and flow sweep experiments. Results showed a shear-thinning behavior with a yield stress of approximately 150 Pa at low shear rate (Figure 4B).

### Finite Element Models of the Peptide-Based Ink

#### Extrusion Process

**Figure 5:**
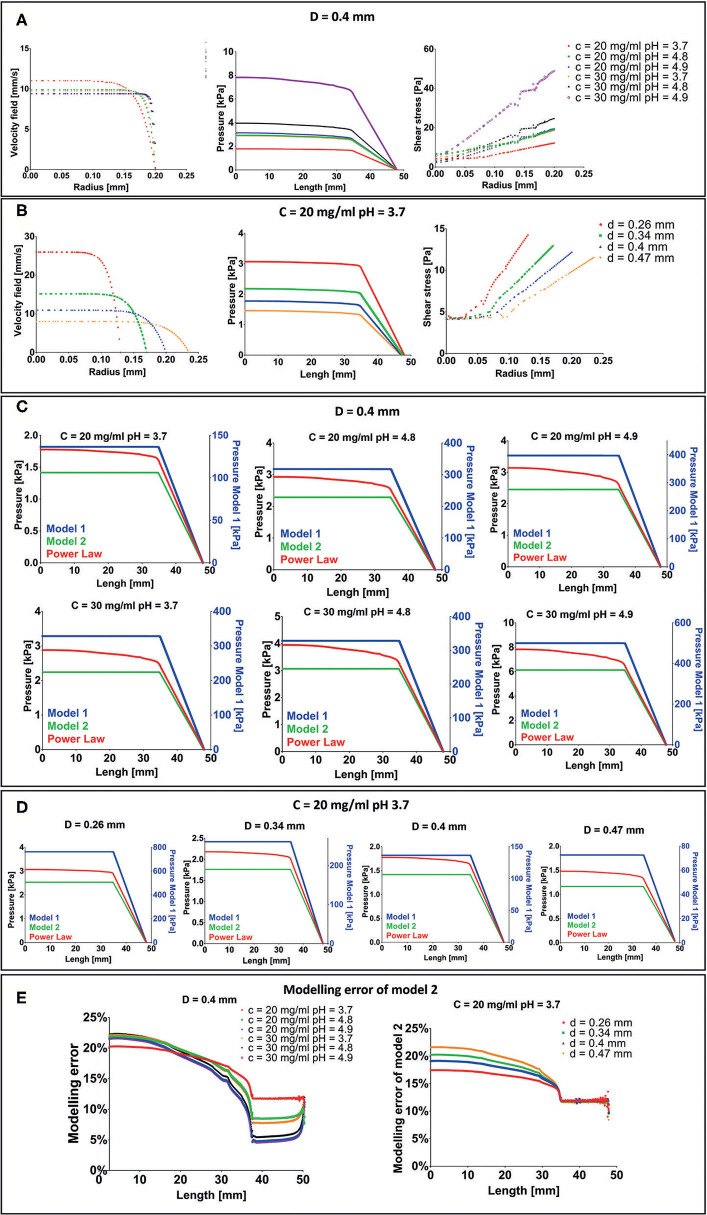
Results of extrusion process finite element (FE) simulation. **(A)** Velocity field, pressure drop, and shear stress for each F9 hydrogel pH and concentration with a needle diameter of 0.4 mm. **(B)** Velocity field, pressure drop and shear stress for each needle diameter when the F9 hydrogel with concentration = 20 mg/ml and pH equal to 3.7 flows in the needle. **(C,D)** Difference in pressure drop was evaluated with three different rheological models, plotted vs. the extrusion system length (syringe + needle) for each combination of hydrogel pH, concentration, and needle diameter. **(E)** Modeling error of model 2 with respect to power law model in the evaluation of the pressure drop in the extrusion system (syringe + needle).

F9 hydrogel extrusion process was simulated by FE models. A piston-driver extrusion-based 3D bioprinter equipped with a cylindrical needle was implemented. F9 hydrogels were described as non-Newtonian fluids by the power law reported in Equation (1). The velocity field and the shear stress were plotted vs. the needle radius at the outlet of the printing needle, whereas the pressure drop was plotted vs. the needle length, for each F9 hydrogel concentration and pH (Figure 5A) and for each needle diameter (Figure 5B). As expected, the flow shows a shear-thinning behavior with a distinct plug-like region in the inner part of the needle, which exceeds the 70% of the section. The radius of the plug-like region increases with *n*, the exponent in Eq. 1, ranging from 76.5 to 90.97% of the section, when *n* is equal to 0.12 and 0.02, respectively. Similarly, the radius of the plug-like region increases with the nozzle radius, ranging from 72.95 to 77.17% of the section, when the nozzle radius is equal to 0.13 and 0235 mm, respectively.

Pressure drop in the system is in the order of magnitude of the kPa and increases with *K* and *n* and decreases with the needle radius. The same behavior was observed for the shear stress. Notably, shear stress was <60 Pa for each simulated condition.

Since the maximum pressure that the piston can apply to the biomaterial ink is 10 MPa (*Bioprinting of Scaffolds*), all the simulated configurations will lead to a successful extrusion process.

Three different rheological models (described in *Pressure Drop Analysis With Different Rheological Models*), with an increasing request of computation power, were then compared to understand the modeling error: Model 1: Newtonian model with viscosity equal to K; Model 2: Newtonian model with viscosity set as the viscosity the F9 hydrogel has at the average shear strain (Equation 3); and Model 3: Power law (Equation 1). Pressure drops vs. extrusion system length (syringe + needle) are shown in Figures 5C,D. In model 1, the pressure drop is two orders of magnitude higher than power law model and model 2. Differently, in model 2 and in the power law model, the pressure drop has the same order of magnitude (~kPa). In particular, model 2 underestimates the pressure drop with respect to the power law model of about 17–23% in the syringe and decreases toward the needle, where it is between 5 and 12% (Figure 5E). This difference is the result of how we have evaluated the $\dot{\gamma }$, which is similar between the two models in the needle part of the extrusion system. The modeling error at the top of the syringe negligibly increases with *n*, ranging from 20.24 to 22.23% when *n* is equal to 0.12 and 0.02, respectively. Similarly, a negligible increase in the modeling error at the top of the syringe, ranging from 17.43 to 21.56%, was recorded when the radius is equal to 0.13 and 0.235 mm, respectively.

#### Mechanical Stability

**Figure 6:**
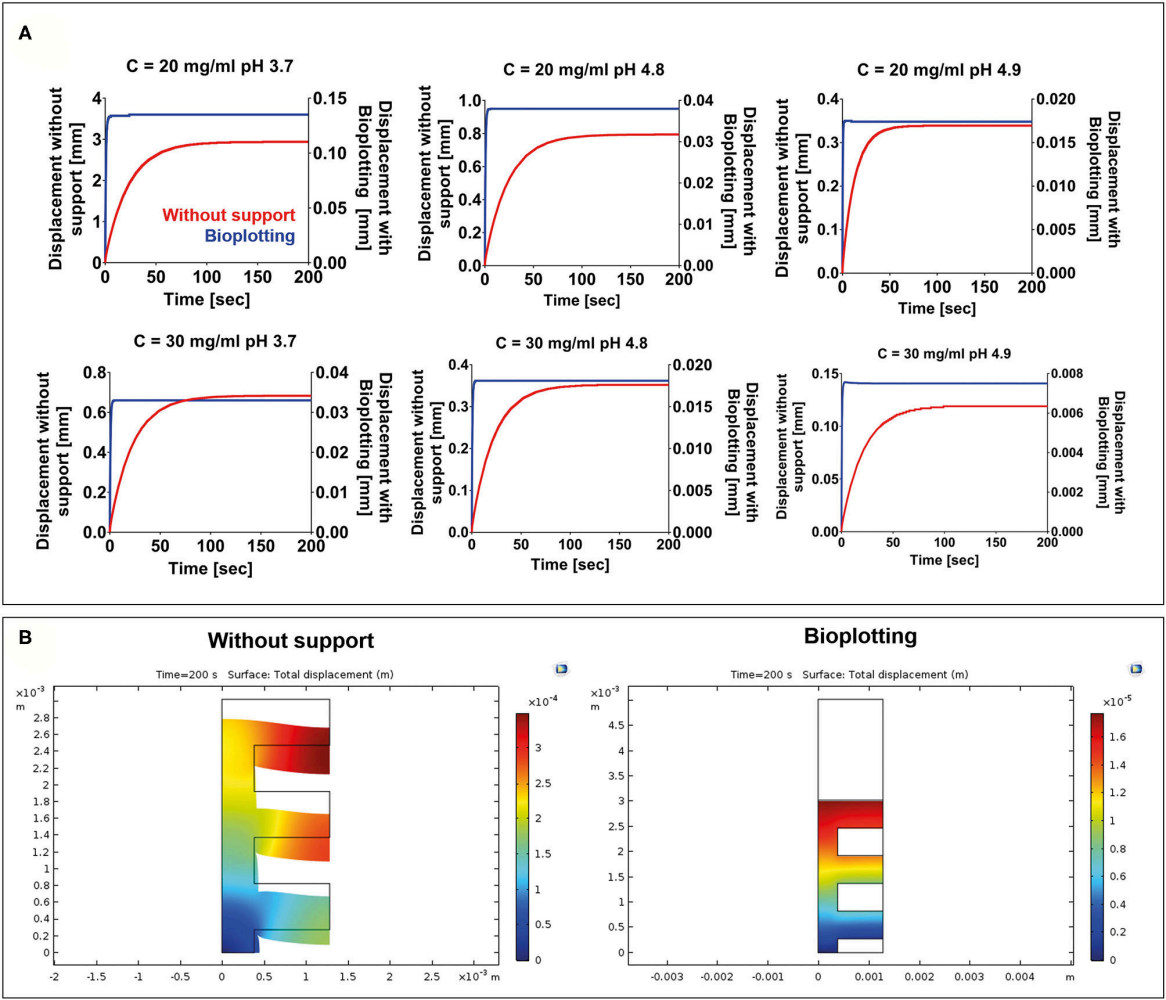
**(A)** Displacement vs. time for each F9 hydrogel concentration and pH. For each combination, two situations were simulated: (1) 3D bioprinting directly on the build plate without any stabilizing strategy (red) and (2) 3D bioprinting into a sacrificial support material (bioplotting, blue). As expected, the presence of the support material substantially decreases the collapse of the scaffold, thus allowing the fabrication of a scaffold with an interconnected pore network. **(B)** A surface plot highlighting the collapse of the scaffolds at the end of the simulation (200 s) when the scaffold is bioprinted without any support and when the bioplotting technique is used. For brevity, only the plots of the F9 hydrogel characterized by a concentration equal to 20 mg/ml and a pH of 4.9 are shown in the figure.

FE simulations were carried out to evaluate the collapse of a 3D-bioprinted wood-pile scaffold due to the gravity force. Two different situations were simulated: (1) the scaffold is bioprinted directly on the build plate without the use of a stabilizing strategy; and (2) the scaffold is bioprinted into a sacrificial support material (bioplotting). Both the scaffold and the sacrificial support material were described by a Kelvin–Voigt lumped parameter model. The displacement over time of the point A (Figure 2) of the scaffold was analyzed for each F9 hydrogel pH and concentration and compared between models 1 and 2 (Figure 6A). As expected, when no support material is used, a higher displacement is registered, ranging from 2.94 to 0.12 mm, which corresponds to a strain going from of 90 to 4% of the structure height, respectively. The deformation of the structure is reduced with the increase of F9 hydrogel concentration and pH. At low pH and concentration (C = 20 mg/ml and pH = 3.7), a complete collapse of the structure is observed. Differently, when the bioplotting technique is used, and the scaffold is bioprinted into the PVA-PVP copolymer, the strain is significantly reduced and negligible, ranging from 3.5 to 0.25%, which corresponds to the displacement of the point A between 0.13 and 7.5 ^*^ 10^−3^ mm (Figure 6B).

### Bioprinting Line Characterization

**Figure 7:**
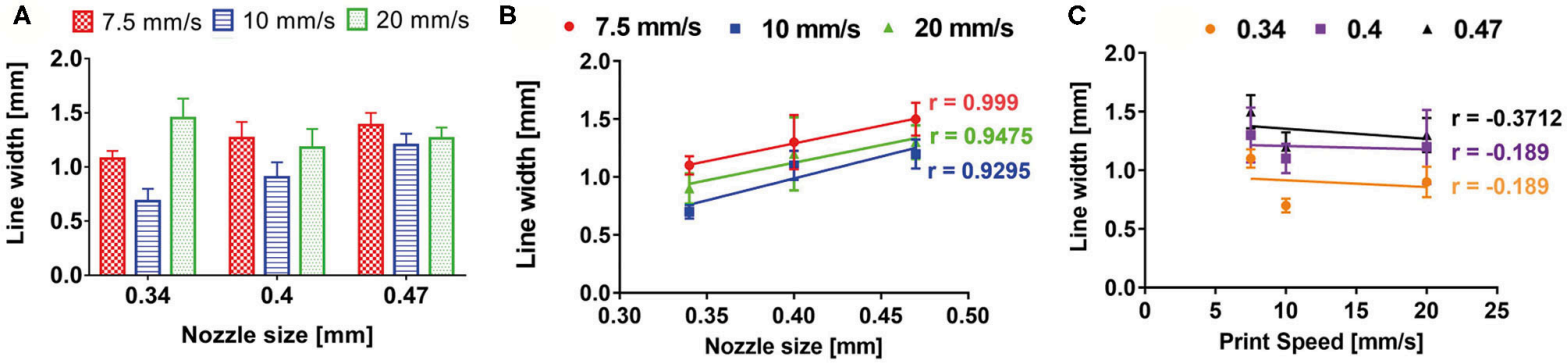
**(A)** Bioprinted line width characterization according to print speed and nozzle size. Pearson correlation coefficient revealed a high correlation between printed line width and nozzle size **(B)**, whereas no correlation was shown between printed line width and print speed **(C)**.

A monolayer snake-like structure (Figure 3B) was bioprinted setting up three different values for the nozzle diameter and the print speed (nine combinations in total). For each combination, the average value of line width was calculated (Figure 7A). The Pearson correlation coefficient was used to identify linear correlation between printed line width and print speed and between printed line width and nozzle size. The analysis showed that the printed line width is highly correlated with the nozzle size (r > 0.9 for each printing velocity) (Figure 7B). Differently, the printed line width is not correlated with the print speed (|r| ≤ 0.37, for any nozzle radius) (Figure 7C).

These results are a direct consequence of the piston-based extrusion and extrusion parameters of the slicing software Slic3r® (developed for FDM 3D printers), which is able to impose a flow rate for keeping the line width constant and proportional to the needle diameter, regardless of the printing speed or the material rheology.

### Bioprinting of Complex Structures

**Figure 8:**
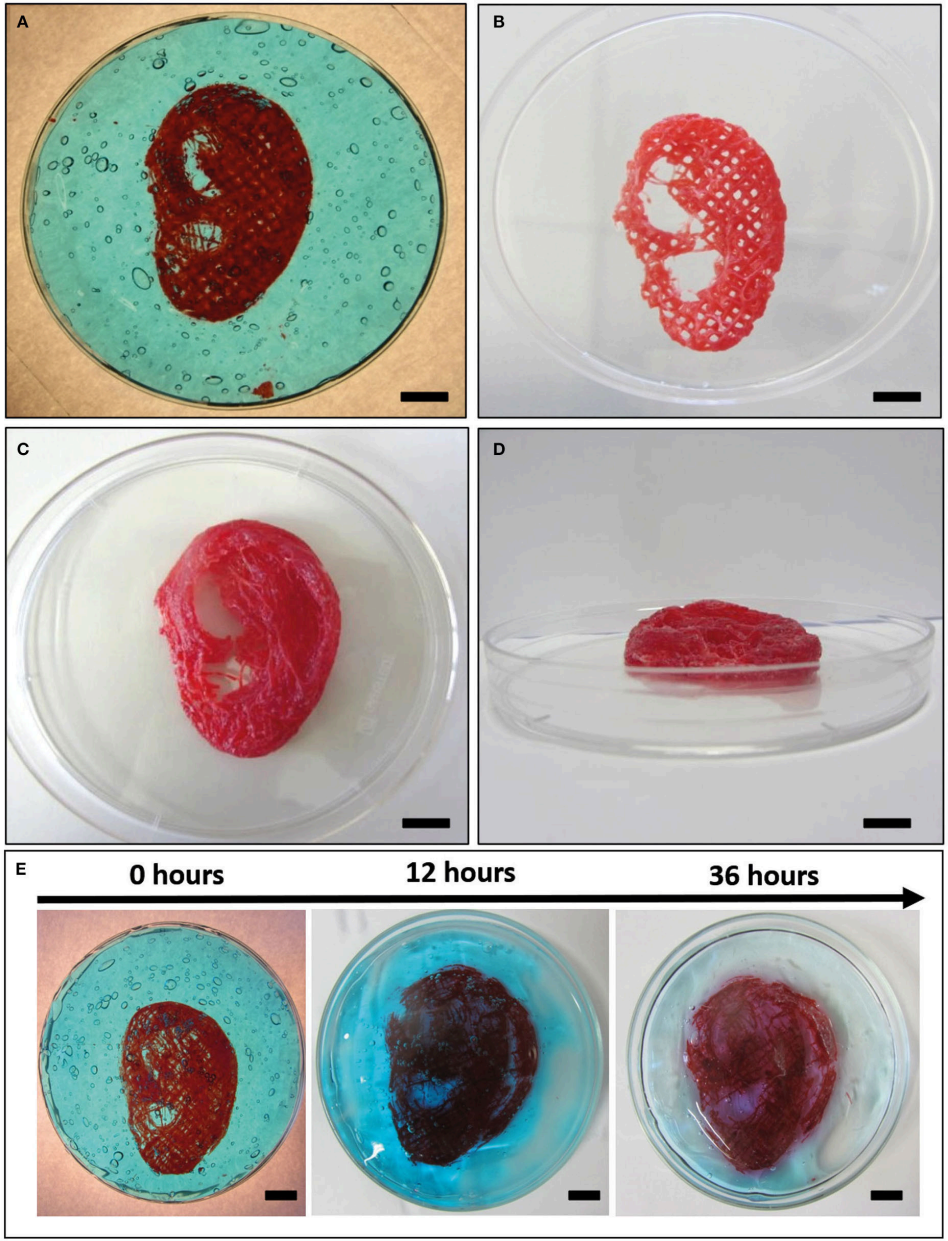
Bioplotting into the poly(vinyl alcohol)–poly(vinyl pyrrolidone) (PVA-PVP) copolymer allowed to bioprint ear-shaped scaffolds with clinically relevant dimensions (~40 × 30 × 20 mm^3^) and several protrusion planes **(A)**. Two self-supporting scaffolds without and with perimeter were successfully bioprinted with 0.77-mm nozzle diameter (**B–D**, respectively). **(E)** When a 0.44-mm needle is used, the bioprinted lines are not solid enough to support the weight of the whole structure. Thus, gradual dissolution of the supporting material in deionized water is accompanied by structural breakdown of the ear-shaped scaffold in time. Scale bar = 10 mm.

A human ear-shaped structure with a complex pore network and several protrusion planes was successfully bioprinted in the PVP-PVA copolymer (Figure 8). Three models were tested (Table 4). After the printing process, the structures were retrieved from the sacrificial support material by immerging them in deionized water for 48 h. The structures that were 3D bioprinted with a 0.77-mm nozzle completely maintained their shape and their structural integrity (Figures 8B–D) when the support material was completely removed. Differently, the structure that was 3D bioprinted with a 0.47-mm nozzle gradually collapsed and broke down as the support material dissolved in deionized water, as shown in Figure 8E.

## Discussion

In this work, we first synthetized and characterized a new β-sheet forming peptide (i.e., as F9 hydrogel), and then we successfully 3D bioprinted a volumetric scaffold with clinically relevant size and several overhang features, as a proof of concept of the use of the F9 hydrogel in bioprinting and TE applications.

The entire 3D bioprinting process (extrusion process and mechanical stability over time of bioprinted scaffolds) was simulated by FE modeling. Thus, thanks to the implementation of robust FE simulations, the trial and error approach typical of 3D bioprinting was minimized.

First of all, rheological analysis of the F9 hydrogels was performed. They possessed a shear-thinning behavior (Figure 4A), which was independent of the peptide concentration and pH. This behavior is well established among β-sheet forming peptides, and therefore, it was also expected for the F9 hydrogels tested in this study (32, 33). As it has been proposed by Schneider and Pochan, in fact, β-sheet-forming peptide hydrogels under shear are fractured into isotropic and disconnected domains of sub-micron size (>200 nm), which slide on each other's surface, allowing gel to flow (32). The shear-thinning behavior is also accompanied by a fast recovery of the hydrogel, since once the shear is removed, hydrogel domains immediately percolate at their boundaries, allowing a fast recovery of initial shape and integrity (32). In fact, we previously shown recovery of FEFKFEFK (F8) systems in >5 min, highlighting the suitability of this class of hydrogels for injectable and printable strategies (29).

Afterwards, FE simulations were performed to analyze the entire extrusion-based 3D bioprinting process, divided into the extrusion of the material from the extrusion system (needle + syringe) and into the mechanical stability overtime of a wood-pile scaffold after material deposition.

Several studies already focused on the importance of simulating the extrusion of the biomaterial ink/bioink to predict the behavior of the material during the bioprinting phase (13, 36, 37).

In this study, in the extrusion process simulation, a piston-driven extruder was implemented. This system has several advantages in comparison with pneumatic-driven extruder. For example, in piston-driven extrusion systems, a constant volumetric flow is maintained and directly controlled by the syringe piston (53). This implies that the average outlet velocity does not depend on the material rheological properties but only on the geometry of the system.

In the simulations, the flow of the hydrogels through the dispensing extrusion system (syringe + needle) was analyzed as the laminar flow through a pipe of a non-Newtonian fluid, which is described by the power law model of Ostwald and de Waele (Equation 1). Due to the high shear-thinning behavior of the F9 hydrogels (*n* ranging from 0.12 to 0.02), a plug-like region was observed in the inner part of the needle, exceeding the 70% of the radius (Figures 5A,B). This implies that 70% of the material is actually not sheared, acting as a solid-like material. Moreover, the maximum pressure needed to extrude is 8 kPa, far below the maximum pressure that the piston can apply (10 MPa). All the combinations of F9 hydrogels and needle diameters have led to a successful extrusion. The quite low value of the pressure needed to extrude the F9 hydrogel is due to its shear thinning behavior that leads to a decrease of the viscosity with the shear rate. In fact, if a Newtonian model is applied, with the viscosity equal to *K*, a higher pressure is reached (Figure 5C). The power law model accurately describes shear thinning fluids; however, it is more complicated than Newtonian model and computationally more expensive. Therefore, we defined another Newtonian model (model 2) that has a constant viscosity set as the viscosity of the power law model at the average shear rate the hydrogel will be subjected in the needle. This model underestimates (Figure 5E) the pressure of the power law model of a maximum of 22.23% at the inlet of the syringes. The differences arising from different rheological properties of the material and from different needle diameter can be considered negligible. These results can be extremely useful to quickly estimate the pressure drop in an extrusion system when a non-Newtonian material is used. As a matter of a fact, the Newtonian model 2 could be used to easily calculate the pressure drop, and then a corrective factor could be introduced to obtain the correspondent values for the non-Newtonian model.

FE simulations are also an important tool to predict cell damage due to the shear stress inside the needle (13, 38). Li et al. (39) quantified the percentage of cell damage after 3D extrusion-based bioprinting process in relation to the residence time of cells in the needle and to the shear stress cells underwent. The authors showed that when the shear stress is below 80 Pa, the cell damage is negligible, even when the residence time is high (300 s). In our study, the maximum shear stress that is reached inside the needle is around 50 Pa. According to Li et al. study, this will induce negligible cell damage, thus showing the possibility to encapsulate cell in the F9 hydrogel to directly deposit cell-laden hydrogel with the investigated fabrication technique. Moreover, the plug flow behavior and the limited shear stresses observed in this study through FE simulations were also reported by Yan et al. (32) with MAX8 β-hairpin peptide hydrogels, which were forced to flow through a 250-μm capillary. This velocity profile was promising for the injection of viable MG63 cells encapsulated in MAX8 hydrogels compared with cells suspended in buffer solution, since within the plug flow, cells experienced little velocity gradient and therefore minimal shear stress (32). It is important to stress out that rheological data we used in the FE simulation were obtained from F9 hydrogel without cells, but they can be assumed equal to the ones we would obtain testing cell-laden F9 hydrogel. In fact, different studies showed that the presence of human cells in the bioink (even with high cell concentration ≥10^9^ cells/ml) does not change the rheological properties of the biomaterial ink (13, 54, 55).

Then, we performed mechanical FE simulations to predict the mechanical stability over time of a wood-pile scaffold after material deposition. Two different bioprinting strategies were simulated: (1) bioprinting directly on the build plate without any stabilizing strategies and (2) bioprinting into a PVA-PVP copolymer that acts as a sacrificial support material bath. As expected, when the support material is not used, the collapse of the scaffold due to the gravity is marked, ranging from 90 to 4% of the structure height. Notably, when the support material is added, the collapse is negligible for all the F9 hydrogels. This is due to the higher viscoelastic properties of the sacrificial material that acts as a support matrix for the F9 hydrogel structure, during the printing process and after it, avoiding its collapse. In addition, using bioplotting, the time at which the structure reaches the equilibrium point decreases.

F9 hydrogels and the PVA-PVP copolymer were both described by the Kelvin–Voigt lumped parameter model. This is a simple model that accurately described creep phenomena (i.e., deformation when a constant force is applied to a viscoelastic body over the time) (49, 50), such as the collapse of the scaffolds due to the gravity force.

The data that we obtained from fluid dynamics and mechanical simulations highlighted the potential of FE modeling to reduce the trial and error attempts typically used in 3D bioprinting and in identifying the suitable biomaterial ink/bioink, extrusion system geometry, and printing parameters. In addition, time-dependent stability of scaffolds actually gives useful information about the appropriate printing process and whether a stabilizing strategy is needed or not. As a matter of a fact, thanks to the mechanical simulations, we identified that bioplotting the SAPHs into the PVA-PVP copolymer is a suitable 3D bioprinting technique to fabricate 3D structure with interconnected pore network.

In bioplotting, an easy-to-remove, shear-thinning material with yielding behavior is used as support matrix for scaffold manufacturing. In addition, the sacrificial support material flows only if it is submitted to a stress above the critical yield stress; otherwise, it acts like a solid material. Therefore, when the nozzle passes through the support material, the yield stress is exceeded and the support material flows, letting the biomaterial ink settle. When the stress is released, the support material acts like a solid, immobilizing the printed filament and preventing the structure to collapse (1, 44). Recently developed sacrificial materials for 3D bioprinting are jammed granular particles, micelles packed into solid-like phases, and polymer networks with reversible bonds (1, 46). In this study, we exploited a PVA-PVP copolymer as a sacrificial support material to allow 3D bioprinting and settling of F9 hydrogel without structure collapse. This resulted in the possibility to successfully bioprint a human ear-shaped scaffold with *in vivo* dimensions and several protrusion planes. The sacrificial support material was easily removed due to its solubility in water. Exposing the F9 hydrogel to deionized water for 48 h did not affect its structure due to the rapid recover time and self-assembly properties of the material itself.

Coupled together, the results shown in this work suggest that large objects (>5 cm) made of peptide hydrogel as biomaterial ink can be bioprinted without physical collapse and with retention of 3D printed features, just with the aid of a water-soluble external stabilizer, as we showed with PVA-PVP. In addition, the printing parameters of large objects can be estimated *a priori* by studying the rheological fingerprint of peptide hydrogels and by implementing them *via* FE modeling.

## Conclusion

In this study, we implemented FE simulations to analyze the entire process of extrusion-based bioprinting of newly developed β-sheet F9 hydrogels. Notably, not only the extrusion process but also the mechanical stability over time of 3D-bioprinted structures were simulated and analyzed as key and necessary elements to predict the printability of the biomaterial ink. Subsequently, using the information obtained by FE simulation, we successfully 3D bioprinted a human ear-shaped structure with *in vivo* dimensions and several protrusion planes, exploiting bioplotting into a PVA-PVP copolymer, as suggested by the mechanical FE simulations. The approach presented in this work minimizes the trial and error approach typical of 3D bioprinting and allows to determine the printability of a biomaterial ink/bioink, taking into account the printing setup, the printing parameters, and the printing strategy, tuned exclusively on the basis of rheological analysis of the material itself.

## Data Availability Statement

The raw data supporting the conclusions of this article will be made available by the authors, without undue reservation.

## Author Contributions

IC, CL, AS, GV, and CDM: research design. CL and AMS: hydrogel preparation and rheological characterization. IC, CL, ADA, AMS, and CDM: acquisition and analysis of data. IC and AFB: FE simulation. IC, CL, ADA, AS, GV, and CDM: drafting the paper or revising it critically. All authors have read and approved the final submitted manuscript.

## Conflict of Interest

The authors declare that the research was conducted in the absence of any commercial or financial relationships that could be construed as a potential conflict of interest.
